# Supplementary material for: How Can We Reduce Dental Fear in Children? The Importance of the First Dental Visit
Source: Children (Basel). 2021 Dec 9;8(12):1167. doi: 10.3390/children8121167 (PMC8700154; doi:10.3390/children8121167)
Supplement: Supplementary file 1 [file children-08-01167-s001.zip › children-1487091-supplementary.pdf]

QUESTIONNAIRE

1. What is your family's socioeconomic level?

    - Very low
    - Low
    - Medium
    - High

2. What is the highest level of education in the family?

    - No education
    - Less than primary school
    - Primary school
    - Secondary school
    - Baccalaureate
    - University

3. Who lives at home with the child on a regular basis?

    - Only the father
    - Only the mother
    - Both of the parents
    - Other relatives

4. Child's age:…………

5. Child's gender:

    - Male
    - Female

6. At what age did your child first go to the dentist?

    - One year old
    - Two years old
    - Three years old
    - Four years old
    - Five years old
    - Six or more years old

7. How often do you take your child to the dentist?

    - Never gone
    - When there is a problem or pain
    - Every two or three years
    - Once a year
    - Every six months

8. Has the child ever suffered a bad experience at the dentist?

- Yes
- No

(If not, go to question 11)

9. How old was your child when that bad experience occurred?

……………………

10. Had your child gone to the dentist before that day?

- No, never
- Once or twice
- Yes, from three to five times
- Yes, from six to ten times
- Yes, more than ten times

11. After that experience, was your child more nervous the next few times he/she had to go to the dentist?

- No, nothing
- A little more
- Much more
- He has not been to the dentist since

12. How much does your child agree with the following statements?

	Disagree	A little agree	Somewhat agree	Moderately agree	Strongly agree
I feel anxious shortly before going to the dentist.					
I generally avoid to the dentist because I find the experience unpleasant or distressing.					
I get nervous or edgy about upcoming dental visits.					
I think that something really bad would happened to me if I were to visit a dentist.					
My hearth beats faster when I go to the dentist.					
I delay making appointments to go to the dentist.					
I often think about all the things that might go wrong prior to going to the dentist.					
